# Supplementary material for: A Comprehensive Assessment of Ultraviolet-Radiation-Induced Mutations in Flammulina filiformis Using Whole-Genome Resequencing
Source: J Fungi (Basel). 2024 Mar 20;10(3):228. doi: 10.3390/jof10030228 (PMC10971301; doi:10.3390/jof10030228)
Supplement: Supplementary file 1 [file jof-10-00228-s001.zip › Supplementary Material S8/KEGG annotation/out/64381550635650.os/KO/out_map/map04924.html]

KEGG PATHWAY: Renin secretion - Reference pathway


|  |  |
| --- | --- |
| **Renin secretion - Reference pathway** |  |

[
Pathway menu
| Organism menu
| Pathway entry
| Show description
| User data mapping
]

The aspartyl-protease renin is the key regulator of the renin-angiotensin-aldosterone system, which is critically involved in extracellular fluid volume and blood pressure homeostasis of the body. Renin is synthesized, stored in, and released into circulation by the juxtaglomerular (JG) cells of the kidney. Secretion of renin from JG cells at the organ level is controlled by the four main mechanisms: the sympathetic nervous system, the local JG apparatus baroreflex, the macula densa mechanism, and several hormones acting locally within the JG apparatus. Renin secretion at the level of renal JG cells appears to be controlled mainly by classic second messengers, namely cAMP, cGMP, and free cytosolic calcium concentration. While cAMP generally stimulates renin release and the intracellular calcium concentration suppresses the exocytosis of renin, the effects of cGMP in the regulation of the renin system are more complex as it both may stimulate or inhibit renin release.

|  |  |
| --- | --- |
| Reference pathway | 100% |
